# Humic Substances Reduce Inactivation of Alkaline Phosphatase Induced by Ultrasound at Therapeutic Intensities

**DOI:** 10.3390/biom16081087

**Published:** 2026-07-24

**Authors:** Georgii S. Mikhailov, Maria G. Chernysheva, Ivan V. Mikheev, Daria-Maria V. Ratova, Alexander M. Arutuynyan, Gennadii A. Badun, Alexander L. Nikolaev

**Affiliations:** 1 Department of Chemistry, Lomonosov Moscow State University, 119234 Moscow, Russia; georgii.mikhailov@chemistry.msu.ru (G.S.M.); mikheev.ivan@gmail.com (I.V.M.); darmarrat@gmail.com (D.-M.V.R.); badunga@my.msu.ru (G.A.B.); nicmsu@gmail.com (A.L.N.); 2 A.N. Belozersky Research Institute of Physico-Chemical Biology MSU, 119234 Moscow, Russia; alarut@genebee.msu.ru

**Keywords:** alkaline phosphatase, ultrasonication, humic substances, enzymatic calcium phosphate

## Abstract

The present study investigated the influence of ultrasonication and humic substances (HS) on the colloidal-chemical characteristics and enzyme activity of alkaline phosphatase (AP). Specifically, the distribution of AP in immiscible liquid systems, its adsorption at the liquid–liquid interface, and its enzyme activity were examined. The latter was assessed by measuring both the enzyme’s ability to hydrolyze 4-nitrophenyl phosphate and its capacity to catalyze the synthesis of calcium phosphate. Under alkaline pH conditions, HS preserved the enzyme activity of AP during ultrasonication. Ultrasonication did not alter the secondary structure of the protein, and the observed loss of enzyme activity is reversible for both free AP and its mixture with HS. This reversibility was further supported by the enzymatic synthesis of calcium phosphate, which yielded comparable results for both treated and untreated AP samples. The following mechanism of HS action in the AP-HS system is proposed: (1) HS fragments form complexes with AP, resulting in higher enzyme activity; (2) continued ultrasonic treatment leads to a sharp decline in the activity of free AP; and (3) after the ultrasonic treatment is completed, the enzyme activity remains reduced; however, complexes between HS fragments and AP may begin to reform and exert their effect again.

## 1. Introduction

The propagation of ultrasonic waves through a liquid medium induces particle displacement, generating alternating cycles of compression and rarefaction. During rarefaction, when the distance between adjacent molecules exceeds a critical threshold, microbubbles are formed. These bubbles oscillate, growing over successive cycles until they reach an unstable size and implode violently. This implosion, known as cavitation, produces intense local effects, including high shear forces, extreme temperatures, and generation of free radicals [1]. Nowadays, interest in ultrasonication is justified due to its broad potential in various fields [2,3,4,5,6]. Techniques based on ultrasonication serve as a tool for food processors to meet consumer and industry expectations, delivering enhanced product safety and quality while optimizing energy use [7]. It is a promising eco-friendly tool for water purification and the leaching of organometallic compounds from soils [8]. Furthermore, ultrasonication is one of the effective approaches for co-encapsulation of bioactive compounds within protein-based nanodelivery systems [9]. Nevertheless, in some cases, it is necessary to encapsulate proteins, especially enzymes, to safeguard their functional activity. In this aspect, natural biopolymers, such as humic substances (HS), are promising due to their wide distribution in the environment, physical and chemical characteristics, and their own biological activity [10]. While humic substances and enzymes can coexist in both water purification and food disinfection systems, the issue of their mutual influence under ultrasonic treatment has not been previously studied. The effects of ultrasonic treatment on HS and enzymes considered separately can be summarized as follows.

It is critically important to consider the intrinsic properties of the enzyme and the fluid when analyzing the effect of sonication [11,12]. Enzymes can exhibit either complete stability or inactivation; the latter can be mitigated by increasing the process fluid viscosity [11,12,13,14,15]. Research indicates that ultrasound technology can be employed to inhibit browning enzymes, including polyphenol oxidase, peroxidase, and lipoxygenase, in fruits and vegetables. Furthermore, this treatment helps denature harmful pathogens, contributing to enhanced food safety [16]. On the other hand, the activity of some enzymes like pepsin, xylanase, α-amylase, and cellulase can be enhanced by ultrasonic application [16,17,18]. The enhancing effect was primarily attributed to an increased interaction between substrate molecules and enzyme active sites. This was facilitated through several mechanisms: improving mass transfer within the reaction system; degrading the substrate, leading to disruption of its aggregates; increasing the enzyme-substrate interfacial area in liquid–liquid systems; and reducing the substrate’s degree of polymerization, thereby making it more susceptible to enzymatic attack [16,17,18]. For pepsin and cellulase, it was shown that changes in the secondary structure caused by ultrasonication did not result in any significant changes in enzyme activity [19].

As for HS, ultrasonication is a very effective tool for extracting humic and fulvic acids from coals and peat [20], and humic acids from lignocellulose [21]. On the other hand, HS can be affected by ultrasound. Two mechanisms underlie the effect of ultrasonication on humic substances. One mechanism involves the chemical degradation of HS within cavitation bubbles via oxidation. The other is the physical aggregation of HS fragments. The processes are controlled by temperature, pH, conductivity, and redox potential [22]. It should be noted that the cited studies employed ultrasound with a maximum horn power output of 130 W/cm^2^ at 30 kHz [21] or with calorimetric intensities ranging from 6.3 to 42.4 W/cm^2^ at 20 kHz [22].

Given that the mutual interactions between HS and enzymes under ultrasonic treatment remain unexplored, the present study aimed to address this gap. Alkaline phosphatase (AP) was selected as a model enzyme due to its wide distribution across different ecosystems. It is a key enzyme in the phosphorus cycle and is ubiquitously present in environments harboring microorganisms, plants, or animals [23,24]. This widespread distribution ensures direct contact between AP and HS.

In the present study, therapeutic ultrasound at 0.88 MHz and an intensity of 2 W/cm^2^ was applied to induce pronounced effects on both the enzyme and HS [25,26,27]. The authors analyzed the effects of HS and ultrasonication on the colloidal-chemical characteristics of AP, including distribution in the system of immiscible liquids and adsorption at the liquid–liquid interface, as well as its enzyme activity. The latter was characterized by the enzyme’s ability to hydrolyze 4–nitrophenyl phosphate, as well as to catalyze the synthesis of calcium phosphate. Colloidal properties were investigated using a radiochemical approach with tritium-labeled AP and liquid scintillation spectroscopy as a scintillation phase technique [28]. A key advantage of the tritium-based radioactive tracer method is its applicability to complex compounds such as HS, rather than to their analogs. The tritium thermal activation method, also employed in the study, enables the preparation of labeled HS with a uniform distribution of tritium across HS fragments; therefore, the labeled HS retains chemical composition, structure, and properties of the native material [29,30].

## 2. Materials and Methods

### 2.1. Materials

*E. coli* alkaline (purity > 90% as declared by the manufacturer) and Tris-buffer (pH 8.5 at 25 °C; 10 mM Tris–HCl, 100 mM NaCl, 10 mM MgCl_2_, and 1 mM DTT) were obtained from SibEnzyme (Novosibirsk, Russia). To examine the pH dependence of AP properties, buffer solutions at pH 7.0, 8.4, and 9.0 were prepared by titration with HCl and NaOH.

Humic acids were isolated from “Irkutskii gumate” (AGROTECH GUMAT LLC, Angarsk, Russia) and kindly provided by Prof. Perminova (Department of Chemistry, Lomonosov Moscow State University, https://perminovalab.ru/en/, accessed on 23 July 2026). The main characteristics of the HS sample are presented in references [31,32]. In the present study, the HS sample was further characterized by potentiometric titration with 0.1 M NaOH using an automatic titrator 848 Titrino plus (Metrohm, Herisau, Switzerland).

Calcium glycerol phosphate (purity > 98%) was a product of Sigma Aldrich (St. Louis, MO, USA). Tritium-labeled alkaline phosphatase was obtained using the tritium thermal activation method [33,34].

### 2.2. E. coli Alkaline Phosphatase Enzyme Activity Measurement

A Vektor-Best kit (Moscow, Russia) was used for determining the AP enzyme activity measured by the hydrolysis of 4–nitrophenyl phosphate (pNPP) in diethanolamine buffer (pH 10.4, 1.14 M diethanolamine, 0.5 mM MgCl_2_, and 0.1% w. NaN_3_), with the formation of yellow-colored 4–nitrophenol (pNP). All reagents were heated up to 37 °C before mixing. A 5 μL portion of the AP solution was added to 1 mL of 10 mM pNPP. The rate of change in absorbance ($\frac{dA}{dt}$) of pNP was measured at 405 nm using a Hitachi U-5100 spectrophotometer (Tokyo, Japan) in the time-scan mode. Enzyme activity (A) was determined as follows:

(1) $$A=\frac{dA}{dt}\frac{V}{\varepsilon vl}$$

Here, V is the total volume of reagents in the cuvette, v is the probe volume, ε is the apparent molar absorptivity of pNP, and l is the optical path length.

### 2.3. Preparation of Tritium-Labeled Alkaline Phosphatase

The standard method of introducing tritium into organic compounds by the tritium thermal activation method was previously described in reference [33]. A brief procedure developed for alkaline phosphatase is as follows. *E. coli* alkaline phosphatase was concentrated using centrifugal concentrators with a 10 kDa molecular weight cut-off filter membrane (Thermo FS, Waltham, MA, USA) and washed three times with water in the same concentrator to purify protein from the buffer solution in which it was supplied. Then, the protein was diluted in water and evenly applied to the walls of the glass reaction vessel designed for tritium labeling [33], followed by lyophilization.

The reaction vessel was then connected to the device designed for operation with gaseous tritium. After evacuation, it was filled with tritium to a pressure of 0.5 Pa. Tritium atoms were generated on the surface of a tungsten filament placed in the center of the reaction vessel when it was heated by the electric current up to 1810 K for 10 s. During the treatment, the walls of the reaction vessel were cooled with liquid nitrogen to prevent protein decomposition. After the reaction, the residual gas was pumped out, and the protein was dissolved in 0.9% NaCl. The initial radioactivity was measured using liquid scintillation spectrometry. To purify protein from labile tritium and low-molecular-weight by-products, a 7-day dialysis was performed. The final solution was lyophilized, and the solid was washed with toluene. It should be noted that each step was controlled through measuring both radioactivity and enzyme activity. It was found that enzyme activity was preserved during the labeling process, and the specific radioactivity of the final product was 1.8 Ci/g.

### 2.4. Ultrasonication Treatment

**Figure 1:**
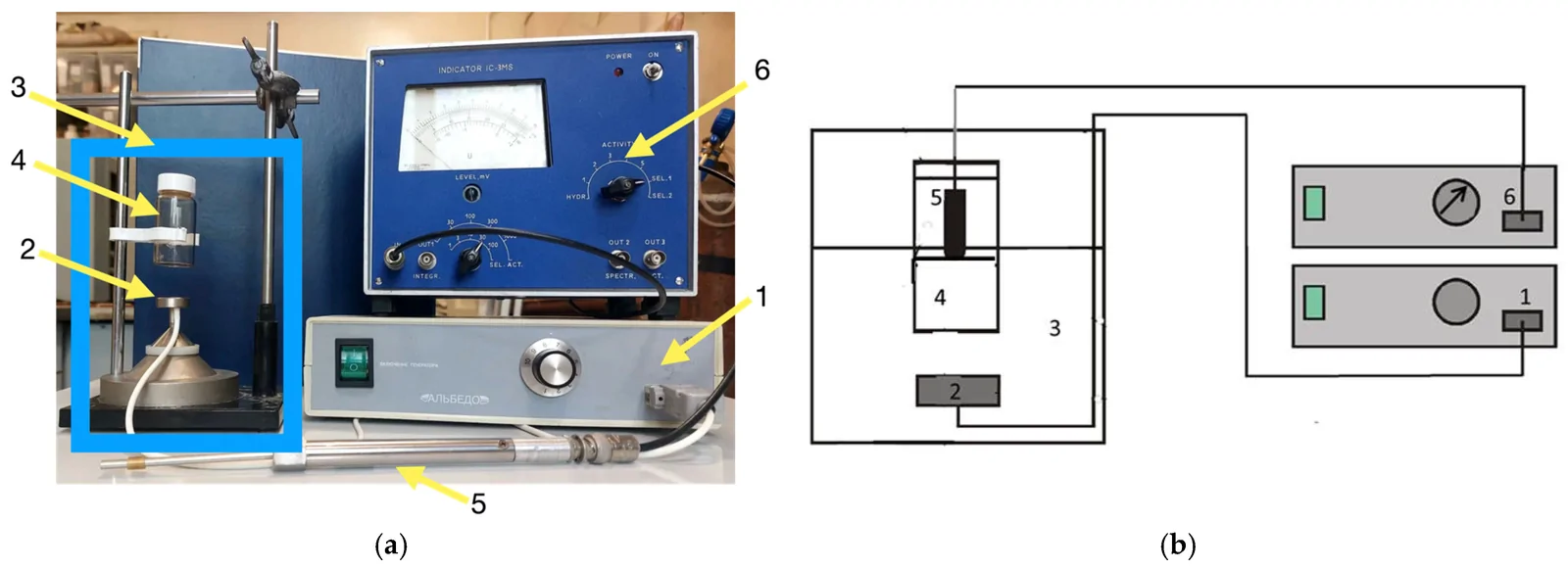
Scheme of the setup for sonication treatment: (**a**) photo and (**b**) diagram. 1—generator “ALBEDOPMP” (Moscow, Russia); 2—ultrasound transducer; 3—thermostat; 4—sample cell; 5—hydrophone; 6—cavitometer IC-3MS (Minsk, Belarus).

Two AP solutions (30 mg/L, specific activity 50 MBq/L) were prepared in Tris-buffer. One of the solutions also contained 10 mg/L HS. These solutions were treated with ultrasound at an intensity of 2 W/cm^2^ and a frequency of 0.88 MHz in a thermostatically controlled (25 °C) cell through a sound-conducting medium. The installation shown in Figure 1 includes an ultrasound source. The sample is placed in a standardized vial within a thermostatically controlled cell during processing.

The volume of the solution was 7 mL. The transducer area (3 cm^2^) ensured processing of the full solution volume. Irradiation was performed in the near field, in cavitation mode. An aliquot of the solution was taken at predetermined intervals to measure the enzyme activity. Sonication was performed continuously for 60 min.

Afterwards, the final enzyme activity was measured, and a 3 × 1 mL sample was taken to study the behavior in a system of two immiscible liquids using the scintillation phase method described in Section 2.5. The residual solution was mixed with a calcium glycerol phosphate solution to assess the influence of sonication on calcium phosphate synthesis [34] (Section 2.6).

After sonication of AP, its CD spectra in the far UV region (190–260 nm) were recorded using a modified Chirascan (Applied Photophysics, Leatherhead, UK) dichrograph at 25 °C in thermostatically controlled, detachable cuvettes with a 0.2 mm path length. The mean molecular weight of amino acid residues was 110 Da; the *Θ*-values were determined as the average value of 6 measurements using Pro-Data Chirascan Software.

### 2.5. Studying the Behavior of Alkaline Phosphatase in the Aqueous–Toluene System

Colloidal-chemical properties of AP before and after sonication, as well as with and without HS, were studied in the aqueous–toluene system using tritium-labeled AP and the scintillation phase method [28,30]. 1 mL of [^3^H]AP solution was placed in a polyethylene scintillation vial (PerkinElmer, Shelton, CT, USA), followed by the addition of 3 mL of a toluene–based scintillator immiscible with water. The two-phase systems were incubated at 25 °C for 7 days. Then the counting rate of tritium beta-radiation was measured for the whole two-phase system (I), as well as in the 1 mL portion of the toluene phase ($I_{1}$), and in the residual two-phase system ($I_{2}$). The protein concentration in the organic phase ($c_{O}$) and at the liquid–liquid interface (Γ) was calculated as follows:

(2) $$c_{O}=\frac{I_{1}}{\varphi a_{sp}V_{1}}$$

(3) $$D=\frac{c_{O}}{c_{W}}$$

(4) $$\Gamma =\frac{I-I_{1}\frac{V_{o}}{V_{1}}}{\frac{1}{2}\varphi a_{sp}S}=\frac{I_{2}-I_{1}\frac{V_{o}-V_{1}}{V_{1}}}{\frac{1}{2}\varphi a_{sp}S}$$

Here, φ is the registra tion coefficient, which relates the counting rate to radioactivity, $a_{sp}$ is the specific radioactivity of [^3^H]AP, $V_{1}$ is the volume of the extracted part of the organic phase (1 mL), *c_W_* is the equilibrium concentration of AP in the aqueous phase, $V_{O}$ is the volume of the organic phase, and S is the liquid–liquid interface area.

After measurement of radioactivity, the interfacial tension was determined using the pendant drop technique. 2 mL of the organic phase was transferred to a quartz cuvette; an aliquot of the solution was taken from the aqueous phase with a syringe (Braun Injection-F), and a drop of 5–6 µL was introduced into the bulk of the organic phase. The surface tension was measured using the OCA 15EC analyzer (DataPhysics, Freiburg, Germany) in the “as fast as possible” mode for 30 min. The calculation of the surface tension was determined by the shape of the droplet according to the Young-Laplace equation using SСA DataPhysics software [35].

### 2.6. Calcium Phosphate Synthesis

A total of 4 mL of sonicated AP and AP + HS solutions each was mixed with 3 mL of calcium glycerol phosphate solution in Tris-buffer, with the resulting concentration being 0.02 M. To mimic the synthesis with untreated AP and AP + HS, 7 mL Tris-buffer solutions containing 0.02 M calcium glycerol phosphate and 30 mg/L [^3^H]AP were prepared; one solution contained 10 mg/L HS. Precipitation began immediately after mixing. The reaction mixtures were incubated at 37 C for 7 days [34]. A barely noticeable brown tint of HS was observed on the surface of the precipitates wherever they were present. After 7 days, the solution was decanted, and the precipitate was washed twice with water. All solutions and the first washings of each sample were measured for tritium activity. The precipitate was dried at 37 °C.

Dry samples of calcium phosphate were analyzed by ATR–FTIR using a Bruker Vertex 70 single-beam IR Fourier spectrometer (Bruker Optics GmbH, Ettlingen, Germany) equipped with a GladiATR™ (PIKE Technologies, Fitchburg, WI, USA) monolithic diamond ATR covering the full spectral range from 4000 to 100 cm^−1^. The number of scans was 64, and the number of background scans was 64. The scanning pitch was set at 2 cm^−1^. The equipment was continuously purged with dry N_2_.

To determine the functional composition, morphology, particle size, and the Ca/P and (Ca + Mg)/P ratios, dry samples of calcium phosphate were analyzed using ATR–FTIR, ICP–AES, SEM and EDX techniques.

An Agilent 720 ICP–AES spectrometer with an axial view (Agilent, Mulgrave, Australia) was used to determine the Ca/P and (Ca + Mg)/P ratios after open vessel digestion with 5% nitric acid; optimization was conducted according to the following protocol [36].

An EDX spectrometer ANAX-30P-B (Thermo Scientific, Waltham, MA, USA) with a specified spectral resolution of 129 eV was used in parallel with a SEM Quattro S (Thermo Scientific, Waltham, MA, USA) microscope at the Shared Research Facility “Electron microscopy in life sciences” at Moscow State University (Unique Equipment: “Three-dimensional electron microscopy and spectroscopy”). Gold-palladium coatings were applied to the samples using vacuum evaporator HUS-5GB (Hitachi, Tokyo, Japan).

### 2.7. Statistical Analysis

Radiochemical studies and enzyme activity measurements were carried out in triplicate. Mean values and standard deviations were calculated using OriginPro (2016) software. Datasets were compared by one-way ANOVA, performed with the same software [37]. Response surface equations were derived using Statistica 10 software [38] by the least-squares method.

## 3. Results

### 3.1. Determining the Optimal Condition for AP and HS Binding

**Figure 2:**
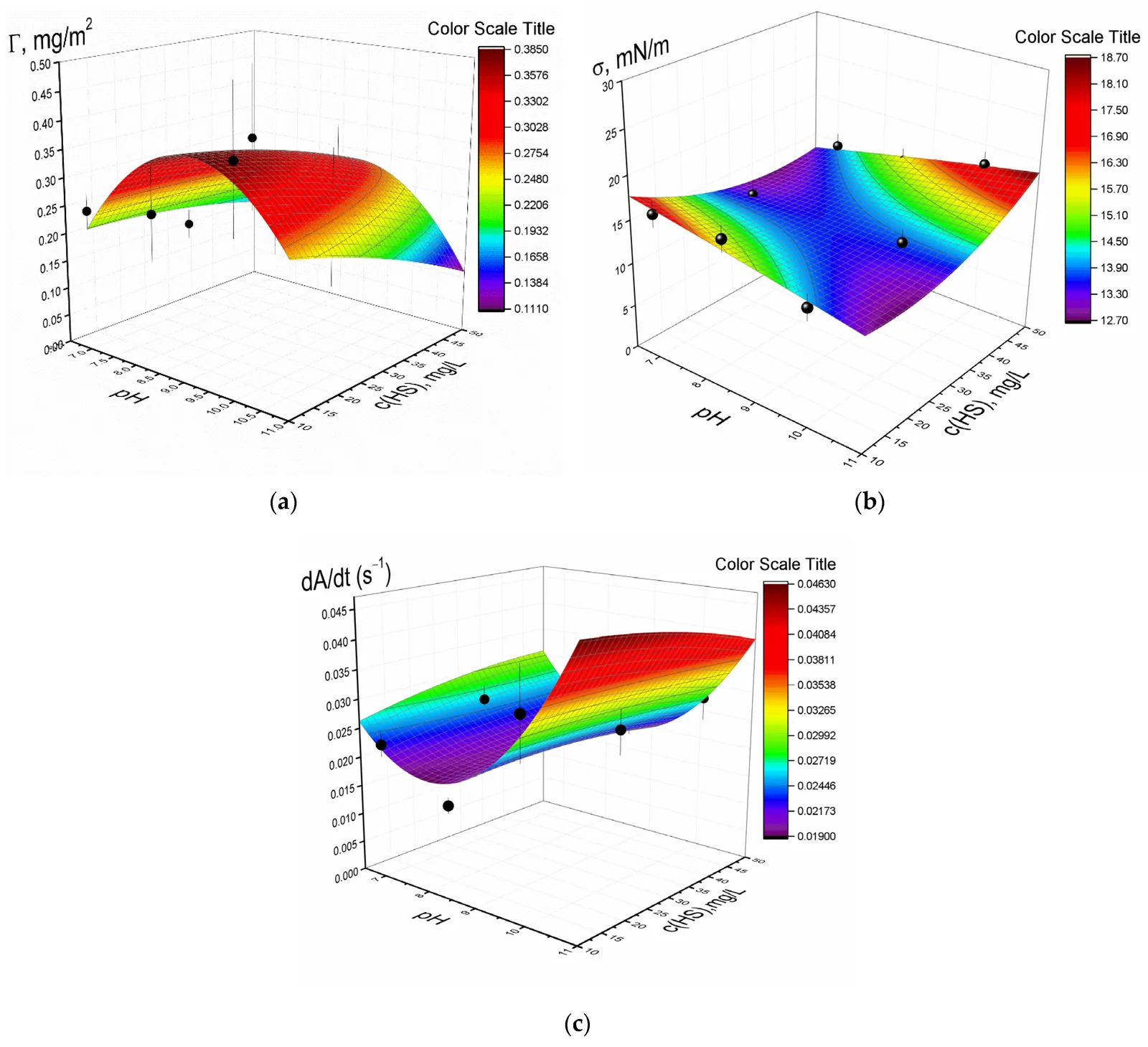
Dependence of adsorption (Γ) (**a**), interfacial tension (σ) at the aqueous–toluene interface for alkaline phosphatase (**b**), and the rate of change in absorbance during the enzymatic transformation of pNPP to pNP (dA/dt) (**c**) on pH and humic substances concentration.

HS have been shown to influence enzyme activity and colloidal behavior [28,39,40,41,42]. In our previous study, we proposed that certain HS fragments can bind to AP, resulting in an increase in enzyme activity [28]. Several parameters can influence the binding process; pH and HS concentrations are considered critically important. In the present study, we changed the pH of the Tris-buffer from 7 to 10 and the HS concentration from 10 to 50 mg/L. A two-factor factorial design was implemented in Statistica 10 software to derive surface equations (Figure 2) describing enzyme activity, AP surface concentration, and interfacial tension as functions of pH and HS concentration.

Surface equations were derived for each response variable (enzyme activity, AP surface concentration, and interfacial tension). The calculated values of the specified pH and HS concentration levels were compared with the corresponding experimental data. It should be noted that each response was measured in triplicate during the experiment. Figure 2 presents data as mean ± standard deviation (*n* = 3, *p* = 0.05). Black dots represent the experimental measurements used to construct the response surface model; the color scale indicates the magnitude of the response variable for each combination of pH and c(HS). Experimental and calculated data, as well as one-way ANOVA analysis, are summarized in Table 1 and Table 2, respectively. Note that the mean square (MS) within the groups for the distribution coefficient was 8 × 10^−7^. Such a very low value indicates that the data in each group are tightly grouped around their average values, reflecting low variability and high reproducibility of the measurements. However, the lack of a significant effect of pH and HS concentration is supported by the ANOVA results: the corresponding *p*-values exceed 0.05 (Table 2).

For all tested properties, a close correspondence between the calculated and experimental data was observed at a significance level of 0.05, confirming the adequacy of the applied model and its suitability for determining the optimal interaction conditions. Based on the two-factor experimental design (with pH and HS concentration as independent variables), the model predicted the optimal interaction conditions as pH 9.25 and an HS concentration of 10 mg/L. These values corresponded to the maximum extent of HS–AP interaction under the model’s validity range, with the results falling within the 95% confidence interval. Consequently, these predicted conditions were employed to study the protective effect of HS against ultrasound exposure, with alkaline phosphatase serving as a model target enzyme exposed to this adverse factor.

### 3.2. Influence of Ultrasonication

**Figure 3:**
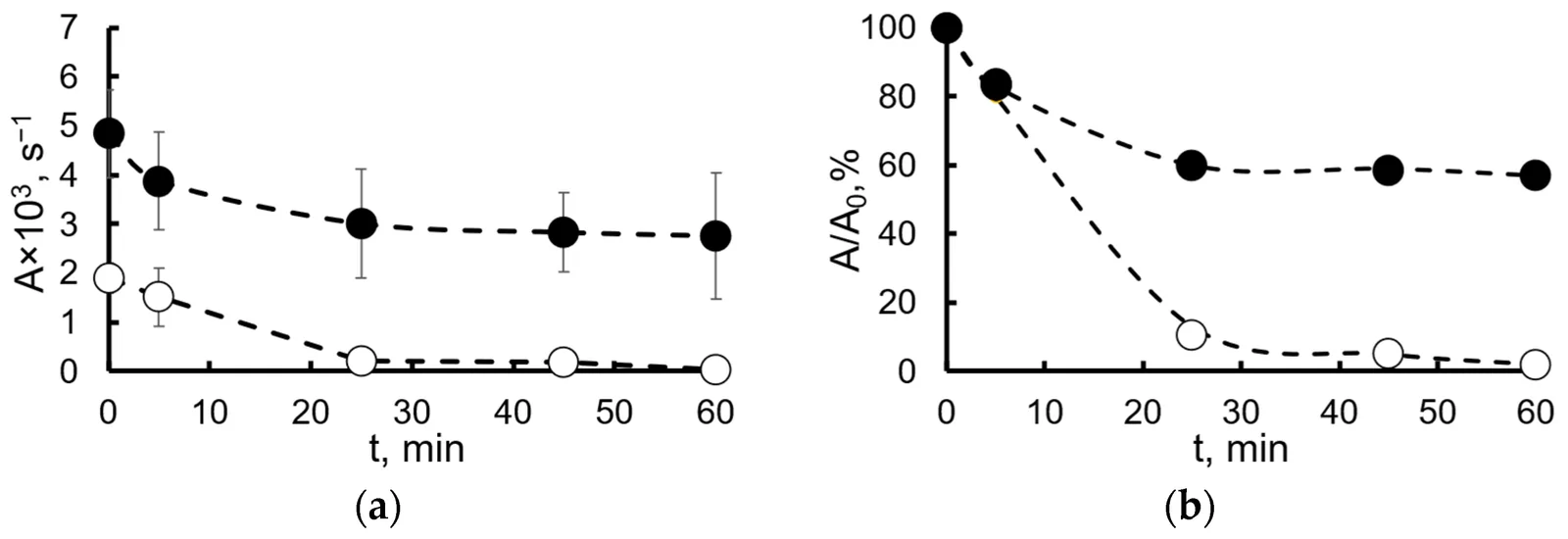
Time dependence of enzyme activity changes during US treatment for AP (open symbols) and AP-HS (black symbols) solutions (*n* = 3, *p* = 0.95). (**a**) Absolute values, and (**b**) relative values.

AP and AP + HS mixture were sampled before US treatment and at times of 5, 25, 45, and 60 min (when US was finished) for enzyme activity measurements. Figure 3 shows the dependence of AP activity with and without HS as a function of US treatment time. The activity data are normalized to the activity of AP before sonication. As described in our previous paper, under the experimental conditions, the addition of HS results in an increase in AP activity up to 3-fold. The reason for the possible activation was discussed in detail in our previous research, where we suggested binding of HS fragments with the AP globule [28].

Within 5 min, the enzyme activity of AP decreased by 20% for both free AP and the AP-HS mixture. Further US treatment led to a decrease in activity up to 90% for free AP, while for the AP-HS mixture, the activity decreased by 40% within 25 min and remained stable thereafter. It should be noted that the absolute values of enzyme activity in the presence of HS were an order of magnitude higher than those for the individual enzyme during ultrasonic treatment for more than 25 min. This effect was no longer offset by a three-fold increase in activity caused by humic acids, but rather indicated their protective role. Therefore, we propose that HS contributes to the preservation of enzyme activity.

Compared with thermal denaturation, which leads to irreversible conversion of the enzyme to an inactive form [43], ultrasonic inactivation of AP can be considered as a reversible or partially reversible process, with enzyme activity recovering over time [44]. This observation confirms that AP does not undergo serious structural changes upon exposure to ultrasound [14,15]. The decrease in enzyme activity measured immediately after treatment may be due to the partially reversible destruction of the metal–organic complex [22] of AP with Mg^2+^, for example, as well as the dissociation of the AP dimer [45]. The presence of a high concentration of Mg^2+^ in the buffer renders such destruction partially reversible. Moreover, the presence of Mg^2+^ and NaCl in the buffer supports stabilization of the dimer structure and, consequently, maintenance of enzyme activity [46,47].

**Figure 4:**
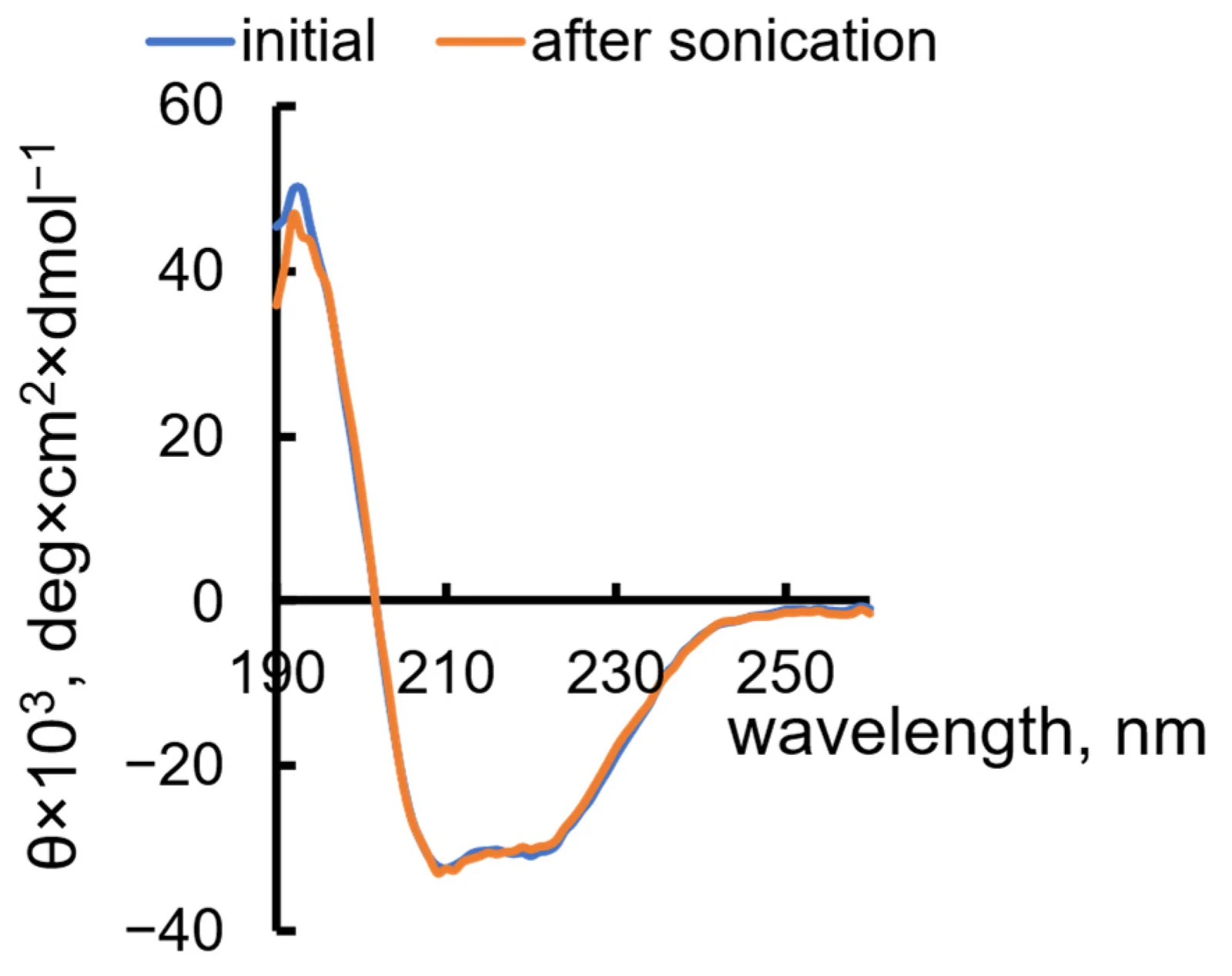
Circular dichroism spectrum of AP before (initial) and after sonication.

Stability of the secondary structure was confirmed by the lack of changes in the circular dichroism spectrum of AP (Figure 4). Negative peaks near 208 and 222 nm indicate a high α-helical content, as also described in the literature [48,49]. The wavelength range was selected based on previous studies, which convincingly demonstrated that characteristic spectral changes are observed in this range, reflecting any restructuring of the secondary structure of the enzyme that may occur during sonication [50,51,52].

It should be noted that the AP distribution coefficient in the aqueous–toluene system and its adsorption at the liquid–liquid interface were determined after 7 days of incubation at 25 °C. This time is necessary to achieve full equilibration of the distribution and adsorption of both AP and HS in the two-phase system. Notably, a 7-day storage of AP at room temperature or at higher temperatures results in enzyme inactivation, which would obscure the role of HS or the ultrasonic treatment. Therefore, enzyme activity was not monitored further; instead, characteristics of the obtained calcium phosphate were measured.

Colloidal characteristics of AP in the aqueous–toluene system with and without HS before and after US treatment are summarized in Table 3.

An increase in the surface concentration of AP and a slight increase in the distribution coefficient after sonication suggest that ultrasonication enhances the surface hydrophobicity of the enzyme [53].

The addition of HS reduces the adsorption value of AP, as previously observed with Tris-buffer (pH 8.5). Sonication results in an increase in adsorption, which becomes similar for free AP and for AP in the mixture with HS. Mechanical degradation, as well as oxidation of HS in cavitation bubbles, occurs in the solution of HS under ultrasound treatment [22]. Such molecular degradation may lead to the formation of low-molecular-weight components that are less surface-active than the initial HS, and thus they do not compete with AP for the interface. This hypothesis was tested in an independent experiment using tritium-labeled HS. Humic acids were subjected to ultrasound treatment under the same conditions, and colloidal-chemical characteristics were obtained for the initial and treated samples, and the treated HS with added AP. For HS, sonication resulted in a decrease in both the distribution coefficient from (5.0 ± 0.3) × 10^−3^ to (2.9 ± 0.2) × 10^−3^, and adsorption from 0.17 ± 0.05 mg/m^2^ to 0.10 ± 0.02 mg/m^2^. Notably, the presence of AP did not affect either the distribution coefficient or adsorption of the sonicated HS.

On the other hand, the main components of HS that can form under ultrasonication treatment—such as phenols and sugars—possess chaotropic activity [54] and therefore contribute to destabilization of the protein structure. Potentiometric titration of the initial HS revealed pKa values close to 4.4 and 9.5, corresponding to carboxyl and phenolic groups, respectively [55,56]. Therefore, in the sonicated solution at pH 9.25, both the degradation mechanisms—electron transfer and hydrogen transfer—are expected. However, given that the latter process is more exergonic, electron transfer is considered preferable [57]. A denatured or partially denatured protein may exhibit a higher capacity for adsorption at the hydrophobic interface.

The formation of calcium phosphate is another factor that influences the enzyme activity of AP. Previously, we showed that an unfavorable environment for the enzyme affects the yield of the resulting phosphate [58].

The synthesis was carried out for 7 days to ensure complete formation of calcium phosphate. Tritium-labeled AP was used to monitor the presence of the protein in the precipitate. Liquid scintillation counting of the decanted solutions and washings revealed that no more than 15% of AP adsorbed onto the resulting calcium phosphate, and this fraction was completely removed during the first washing. It is also expected that HS may be incorporated during calcium phosphate formation and/or adsorbed onto the resulting surface. This is due to the high adsorption capacity of humic substances for hydroxyapatite [59]. Independent experiments with tritium-labeled humic acids showed that under the experimental conditions, about 0.6 mg/g of humic substances remain in the calcium phosphate after washing. Table 4 summarizes the characteristics of calcium phosphate samples synthesized via the enzymatic reaction of AP and the AP-HS mixture, with and without ultrasonication. The Ca/P and (Ca + Mg)/P ratios did not differ significantly among the four samples (one-way ANOVA, *p* > 0.2), showing that neither ultrasonication nor HS altered the stoichiometry of the calcium phosphate.

Enzyme activity was measured after US treatment. The assumption about the restoration of enzyme activity over time is supported, as the mass of the resulting calcium phosphate was similar for AP samples with and without ultrasonication, and lower than in the cases with added humic substances.

**Figure 5:**
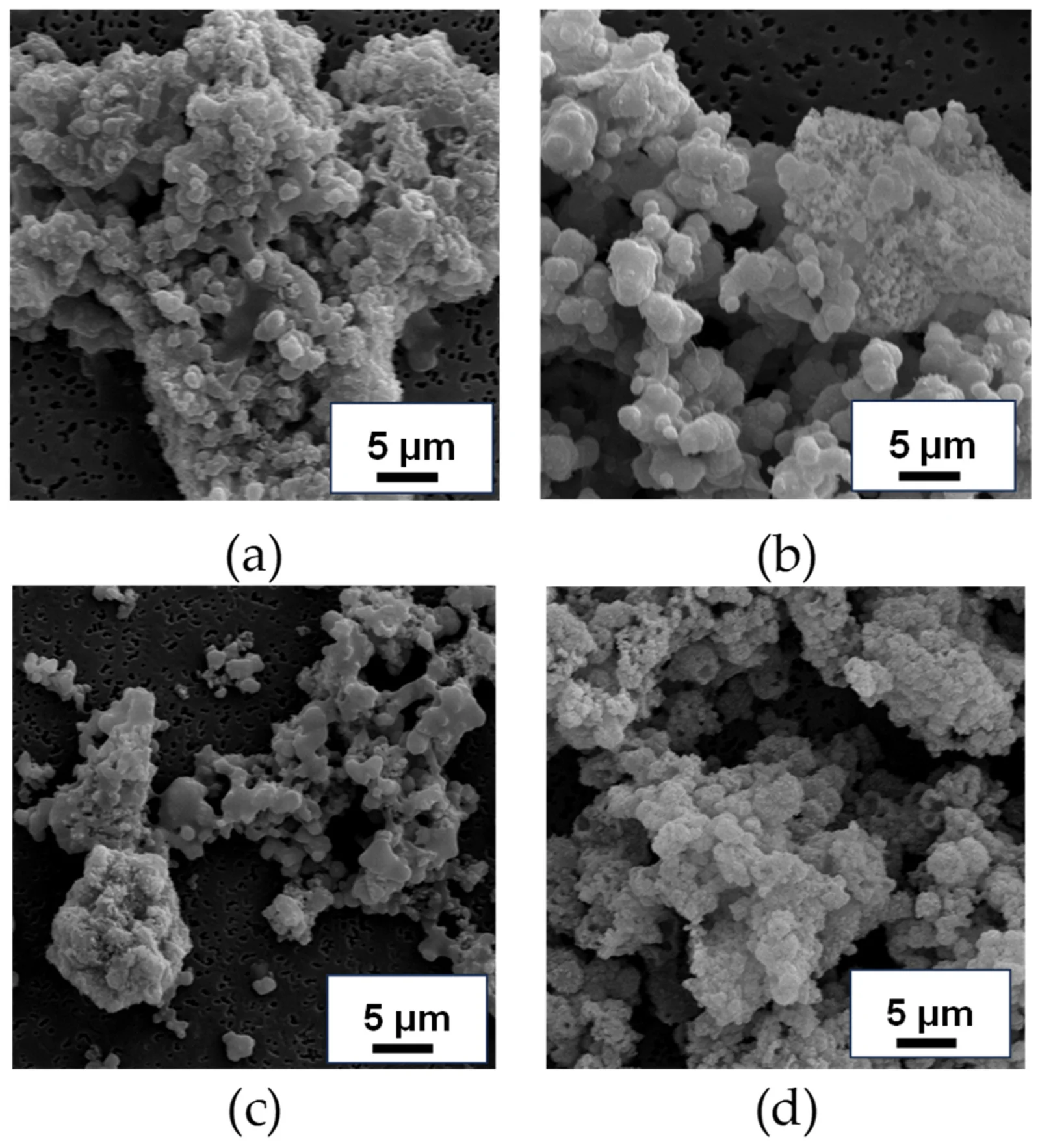
SEM images of calcium phosphates: (**a**)—AP, (**b**)—AP subjected to ultrasonication, (**c**)—AP-HS, and (**d**)—AP-HS subjected to ultrasonication.

SEM images (Figure 5) indicate that the calcium phosphates formed with unaltered AP are porous nanospheres exhibiting a nearly continuous size distribution. Their average radius and its standard deviation were 1.2 ± 0.9, with the largest particles reaching up to 7 μm. The use of unaltered AP led to the formation of polymer-like chains composed of hollow nanospheres—a configuration previously reported only for the early stages of the synthesis [34,60]. US treatment of AP prior to synthesis yields hollow nanosphere clusters ranging from 0.2 to 1.2 μm, with a uniform size distribution and isolated outliers up to 4 μm. It should be noted that the 0.2–0.6 μm size range is reported as typical for hydroxyapatite crystals [61], and particles within this size range were observed in all synthesized samples, providing indirect evidence of their phase composition. Therefore, there is no significant influence of AP pretreatment on activity over the long term.

Similar results were observed for US pretreatment of the AP-HS mixture. Particles of a smaller size were obtained compared to samples without HS (see Table 4), a finding that is also supported by the literature [62,63].

**Figure 6:**
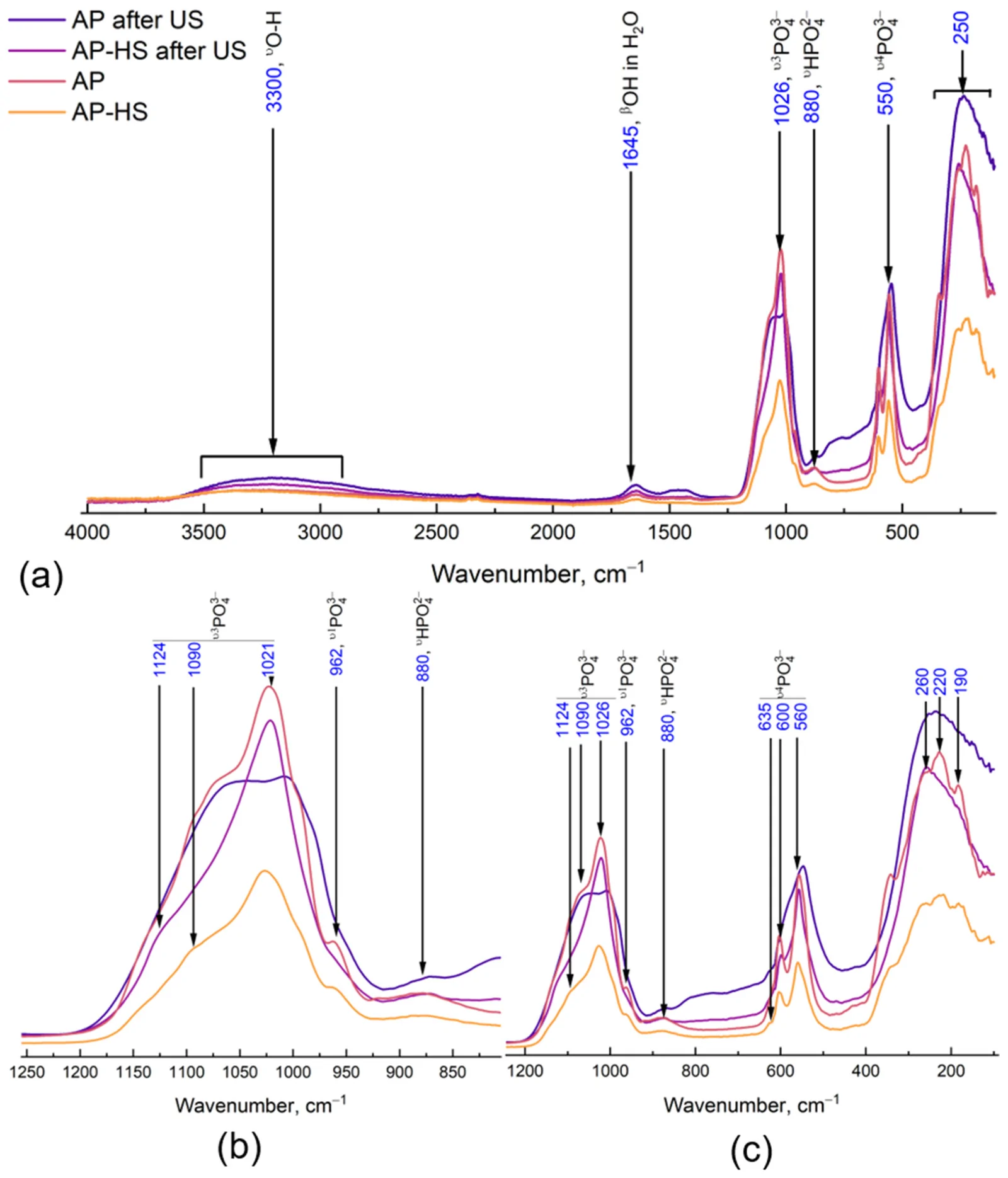
FTIR spectra of calcium phosphates synthesized with free AP, AP-HS mixture, AP subjected to US treatment, and AP-HS mixture subjected to US treatment: (**a**) in range from 4000 to 100 cm^−1^; (**b**) in range from 1250 to 800 cm^−1^; (**c**) in range from 1220 to 100 cm^−1^.

Functional composition of calcium phosphates was determined by FTIR (Figure 6).

The interpretation of FTIR spectral bands for calcium phosphates has been discussed in detail in the recent literature [34]. The spectra of the synthesized calcium phosphate samples are dominated by phosphate vibrations. Bands in the 1100–1000 cm^−1^ region are attributed to the ν_3_ stretching vibrations of PO_4_^3−^ groups, whereas those near 600 and 560 cm^−1^ correspond to the ν_4_ PO_4_^3−^ vibrations. The broad absorption band in the 3600–3000 cm^−1^ region and the band near 1645 cm^−1^ are assigned to hydroxyl groups and adsorbed water, respectively [34].

As follows from Figure 5b, the band near 1020 cm^−1^ becomes less pronounced in the sample obtained with sonicated AP. This spectral feature is commonly observed in non-stoichiometric and poorly crystalline apatites containing HPO_4_^2−^ and CO_3_^2−^ groups [64]. Therefore, its reduced intensity may indicate a lower contribution of non-stoichiometric phosphate environments in this sample.

The possible presence of carbonate species was also considered. Calcium carbonate is typically associated with characteristic bands near 712, 876, and 1420–1470 cm^−1^ [65]. In the present samples, no distinct band was observed near 712 cm^−1^, nor was a well-developed carbonate band envelope evident in the 1420–1470 cm^−1^ region. Therefore, the FTIR data do not support the formation of a separate crystalline calcium carbonate phase. The band near 876–880 cm^−1^ is more consistently assigned to HPO_4_^2−^ groups in non-stoichiometric apatite-like calcium phosphate; however, a minor contribution from carbonate species cannot be entirely ruled out based on FTIR data alone. For this reason, the band at 1400 cm^−1^ may be attributed to atmospheric CO_2_ or traces of adsorbed carbonate, likely due to the high pH of the reaction [58,64,65].

Thus, the less pronounced band near 1020 cm^−1^ in the sample synthesized with sonicated AP suggests a lower contribution of poorly crystalline non-stoichiometric apatite-like environments and a more ordered calcium phosphate structure compared with the other samples.

Elemental analysis was performed by ICP–AES for the bulk sample and by EDX during SEM analysis. The results are summarized in Table 4. EDX showed Ca/P ratios of 1.25–1.30 and (Ca + Mg)/P ratios of 1.37–1.41 at the particle surface, which is consistent with a non-stoichiometric calcium phosphate surface and may indicate octacalcium phosphate-like environments [65,66]. The calculated Mg/(Ca + Mg) ratios from EDX data were 7.8–10.7%, which are comparable to those of Mg-substituted calcium phosphates obtained in our previous enzyme-catalyzed synthesis study [34]. This interpretation is consistent with the FTIR observation of HPO_4_^2−^-containing environments, as evidenced by the band near 876–880 cm^−1^ and by the contribution in the 1100–1000 cm^−1^ region. However, FTIR alone does not permit an unambiguous distinction between poorly crystalline apatite-like and octacalcium phosphate-like phases. Since phase-pure octacalcium phosphate formation requires a narrow synthesis window and dedicated phase confirmation [67], the products are described here as mixed non-stoichiometric calcium phosphates rather than as single-phase hydroxyapatite. ICP–AES analysis of the fully digested bulk samples yielded higher Ca/P ratios of 2.30–2.41, suggesting an additional calcium-rich bulk contribution, possibly related to CaO/Ca(OH)_2_ or another poorly crystalline calcium-rich component [66].

## 4. Discussion

The present study demonstrates that HS preserves enzyme activity during sonication treatment. It was observed that the activity of free AP decreased by 90% after 25 min, while in the presence of HS, the activity decreased by only 40% and subsequently stabilized.

On the one hand, recent findings indicate that the activity of US-treated AP is gradually restored compared to more invasive treatments, such as radiation exposure [42]. It should be noted that in the cited paper, phosphatase enzyme activity decreased by 10% immediately after ultrasound treatment and continued to decline by up to 20% over the subsequent 20 days. Notably, these data are normalized to the untreated sample. So, the result indicates that US-treated phosphatase loses activity faster in comparison with the control sample, whose activity serves as a baseline for normalization. In the present study (Figure 3), the comparison is based on the enzyme activity value prior to processing. Such low enzyme activity (Figure 3) would be insufficient to drive the formation of enzymatic calcium phosphate. The effect of enzyme activity on both the yield and type of calcium phosphate formed was investigated in detail in our earlier work [34,58]. Thus, the characteristics of the resulting calcium phosphate are reflective of the enzyme activity.

However, we observed a comparable chemical yield of calcium phosphate produced by both sonicated and untreated AP, while the particles formed with the US-treated enzyme were smaller and exhibited a crystalline structure. This suggests that the mineral phase had sufficient time to crystallize over 7 days, compared with the sample synthesized by untreated AP. Notably, prolonged incubation is unlikely to affect the precipitate mass, since the formation of amorphous calcium phosphate occurs during the first day. Thereafter, alkaline phosphatase activity ceases, partly due to the presence of calcium cations in the system. The remaining time is spent on the morphological maturation of the precipitate. Therefore, we propose that US treatment results in a reversible or partially reversible effect on enzyme activity.

The addition of HS preserves AP activity and shows the formation of similar calcium phosphates in both treated and untreated samples. This observation may be explained by the enzyme encapsulation by HS. It should be noted that under the experimental conditions, AP is negatively charged; however, there are documented examples of protein encapsulation by HS when both biopolymers carry a negative charge [10]. In the cited paper, the formation of the composite is controlled by hydrophobic interactions. As demonstrated in our earlier work [28], combining a radiochemical approach with molecular docking calculations revealed that hydrophobic components of HS can activate AP by stabilizing the transition state, thereby increasing catalytic activity. Conversely, other fragments may inhibit the enzyme by blocking the active site, while others may remain neutral or exhibit weak interactions. In summary, in the absence of external stress, the net effect of HS is a modest enhancement of enzyme activity. Therefore, the encapsulation of AP by HS is consistent with the preservation of enzyme activity.

By absorbing ultrasonic energy, HS may reduce the direct mechanical impact on the enzyme’s active site. This effect can be considered similar to the protective effect against ultraviolet radiation [68]. By binding to the enzyme, HS reduces direct radiation as well as ultrasound-induced damage to its active site, resulting in significantly less loss of activity compared to free enzymes.

However, this study demonstrates a destructive effect of ultrasound on HS. Changes in the HS colloidal properties after sonication indicate changes in its structural peculiarities. Notably, it is challenging and speculative to use spectrophotometric methods, including HPLC with UV detection, for analyzing structural changes in HS, because in this case we can control aromatic fragments of HS. Light-scattering methods are also unsuitable for HS characterization owing to its high fluorescence. Furthermore, the results obtained with these methods heavily depend upon ionic strength and pH, and thus may not reflect the true state of the sample. In contrast, the colloidal-chemical characteristics mentioned above serve as reliable indicators of the structural state. For instance, we have previously shown that the observed distribution coefficient changes upon micellization [69], as well as in mixtures with molecules such as the well-known surfactant Triton X-100 [70].

Therefore, we can conclude that analysis of the physical and chemical characteristics of the substance is the most appropriate approach in this context. US treatment of HS results in a reduction in both surface activity and hydrophobicity, indicating a decrease in HS hydrophobic and surface-active fragments.

In the present study, US treatment is considered a stress condition. In this case, large HS molecules break down into smaller fragments and the inhibitory components may degrade or lose their activity. The remaining fragments and intact hydrophobic components protect AP from inactivation. Therefore, we propose that the protection mechanism is not merely conformational, but rather physical in nature, involving radical suppression and competition for the surface.

## 5. Conclusions

Under alkali pH, humic substances preserved the enzyme activity of AP during ultrasonication. Ultrasonication leads to the fragmentation of HS molecules into smaller fragments with reduced surface activity compared to the initial material; these fragments are unable to displace AP from the liquid–liquid interface and thus do not compete for it. Ultrasonication does not alter the secondary structure of the protein, and the loss of enzyme activity is reversible for both free AP and its mixture with HS. This is confirmed by the enzymatic synthesis of calcium phosphate, which yields comparable results for both treated and untreated AP samples. The effect of ultrasonication on HS is indirectly confirmed by changes in the colloidal behavior in the liquid–liquid system and changes in the morphological peculiarities of enzymatic calcium phosphate, while the yield and chemical composition remain similar to those of the sample obtained in the untreated AP-HS mixture. Despite the fact that ultrasonication exerts a reversible effect on alkaline phosphatase, the protective properties of humic substances are evident. Therefore, we can suggest the following mechanism of HS action in the AP-HS mixture under ultrasonication:

- Initial complex formation: Some HS fragments form complexes with AP, leading to an increase in enzyme activity [28].
- Prolonged ultrasonication: Continued treatment causes a sharp decline in the activity of free AP.
- Post-treatment recovery: After the ultrasonic treatment ceases, enzyme activity remains reduced; however, complexes between HS fragments and AP may begin to reform and exert their effect again.

## Figures and Tables

**Table 1:** Experimental and calculated values of AP adsorption capacity (Γ), interfacial tension (σ) at the aqueous–toluene interface, and the rate of absorbance change (dA/dt) during the enzymatic transformation of pNPP to pNP under varying pH and HS concentrations.

Conditions		Property Value					
pH	c(HS), mg/L	Γ, mg/m^2^		σ, mN/m		dA/dt, s^−1^	
		Experiment	Calculation	Experiment	Calculation	Experiment	Calculation
7	10	0.25	0.22	16.70	17.21	0.023	0.022
7	30	0.17	0.20	13.50	13.34	0.026	0.025
7	50	0.11	0.15	14.40	14.42	0.024	0.025
8.4	10	0.28	0.36	17.01	15.68	0.016	0.020
8.4	30	0.36	0.33	12.97	13.60	0.023	0.020
8.4	50	0.27	0.27	15.15	16.00	0.020	0.019
10	10	0.39	0.36	13.41	14.18	0.034	0.031
10	30	0.25	0.31	14.22	13.69	0.026	0.030
10	50	0.18	0.23	17.72	17.81	0.027	0.026

**Table 2:** One-way ANOVA results for the experimental values of adsorption capacity (Γ), interfacial tension (σ), and the absorbance change rate (dA/dt).

Property	Source of Variation	Sum of Squares (SS)	df	Mean Square (MS)	F	*p*-Value
Γ, mg/m^2^	Between Groups	2.5 × 10^−3^	1	2.5 × 10^−3^	0.656	0.4297
	Within Groups	6 × 10^−2^	16	3.7 × 10^−3^		
	Total:	6 × 10^−2^	17			
σ, mN/m	Between Groups	0.170	1	0.17	0.110	0.744
	Within Groups	24.71	16	1.54		
	Total:	24.88	17			
dA/dt, s^−1^	Between Groups	8.9 × 10^−7^	1	8.9 × 10^−7^	0.010	0.921
	Within Groups	1.4 × 10^−3^	16	8.9 × 10^−5^		
	Total:	1.4 × 10^−3^	17			

Note: The significance level was set at α = 0.05. ANOVA was performed on experimental data only; calculated values were used for model validation and are reported in Table 1.

**Table 3:** Distribution coefficient (***D***) of AP at the aqueous–toluene interface and its adsorption at the liquid–liquid interface (***Γ***) for free AP and in the presence of HS, without and after sonication (*n* = 3, *p* = 0.95).

Sample	*D*		*Γ*, mg/m^2^	
	Without Sonication	With Sonication	Without Sonication	With Sonication
AP	(5.5 ± 0.4) × 10^−3^	(6.5 ± 0.4) × 10^−3^	0.52 ± 0.12	0.74 ± 0.19
AP-HS	(5.8 ± 0.4) × 10^−3^	(6.3 ± 0.4) × 10^−3^	0.29 ± 0.10	0.81 ± 0.18

**Table 4:** Characteristics of calcium phosphate samples synthesized via the enzymatic reaction of AP and the AP-HS mixture, with and without ultrasonication (*n* = 4, one-way ANOVA, *p* > 0.2).

Parameter	AP		AP-HS	
	Without Sonication	With Sonication	Without Sonication	With Sonication
Enzyme activity (*), U	45	0.1	78	7.2
Mass (**), mg	10.0	12.6	15.9	15.4
Average radius (***), nm	1200 ± 900	470 ± 240	560 ± 60	490 ± 360
Ca/P (EDX)	1.27 ± 0.03	1.25 ± 0.03	1.30 ± 0.03	1.25 ± 0.03
Ca/P (ICP–AES)	2.39 ± 0.24	2.32 ± 0.23	2.41 ± 0.24	2.30 ± 0.23
(Ca + Mg)/P (EDX)	1.40 ± 0.04	1.37 ± 0.04	1.41 ± 0.04	1.40 ± 0.04
(Ca + Mg)/P (ICP–AES)	2.50 ± 0.25	2.40 ± 0.24	2.51 ± 0.25	2.41 ± 0.24

(*) EA was measured before synthesis; (**) weighing accuracy is 0.1 mg; (***) an average radius calculated from SEM images ± standard deviation.

## Data Availability

The original contributions presented in the study are included in the article, further inquiries can be directed to the corresponding author.

## Abbreviations

The following abbreviations are used in this manuscript:

US	Ultrasound
AP	Alkaline phosphatase
HS	Humic substances
pNPP	4–nitrophenyl phosphate
pNP	4–nitrophenol
FTIR	Fourier-transform infrared spectroscopy
ICP–AES	Inductively coupled plasma atomic emission spectroscopy
EDX	Energy-dispersive X-ray spectroscopy
SEM	Scanning electron microscopy
